# A simulation-based phantom model for generating synthetic mitral valve image data–application to MRI acquisition planning

**DOI:** 10.1007/s11548-023-03012-y

**Published:** 2023-09-07

**Authors:** Chiara Manini, Olena Nemchyna, Serdar Akansel, Lars Walczak, Lennart Tautz, Christoph Kolbitsch, Volkmar Falk, Simon Sündermann, Titus Kühne, Jeanette Schulz-Menger, Anja Hennemuth

**Affiliations:** 1 Institute of Computer-Assisted Cardiovascular Medicine, Deutsches Herzzentrum Der Charité (DHZC), Berlin, Germany; 2 Charité–Universitätsmedizin Berlin, Corporate Member of Freie Universität Berlin and Humboldt Universität Zu Berlin, Berlin, Germany; 3 Department of Cardiothoracic and Vascular Surgery, Deutsches Herzzentrum Der Charité (DHZC), Berlin, Germany; 4 Fraunhofer MEVIS, Berlin, Germany; 5 Physikalisch-Technische Bundesanstalt (PTB), Braunschweig and Berlin, Germany; 6 DZHK (German Center for Cardiovascular Research), Partner Site Berlin, Berlin, Germany; 7 Department of Cardiology and Nephrology, Helios Hospital Berlin-Buch, Berlin, Germany; 8 Department of Diagnostic and Interventional Radiology and Nuclear Medicine, University Medical Center Hamburg-Eppendorf, Hamburg, Germany

**Keywords:** Image simulation, Mitral valve, Modeling, Segmentation, Cardiac phantom, Magnetic resonance imaging

## Abstract

**Purpose:**

Numerical phantom methods are widely used in the development of medical imaging methods. They enable quantitative evaluation and direct comparison with controlled and known ground truth information. Cardiac magnetic resonance has the potential for a comprehensive evaluation of the mitral valve (MV). The goal of this work is the development of a numerical simulation framework that supports the investigation of MRI imaging strategies for the mitral valve.

**Methods:**

We present a pipeline for synthetic image generation based on the combination of individual anatomical 3D models with a position-based dynamics simulation of the mitral valve closure. The corresponding images are generated using modality-specific intensity models and spatiotemporal sampling concepts. We test the applicability in the context of MRI imaging strategies for the assessment of the mitral valve. Synthetic images are generated with different strategies regarding image orientation (SAX and rLAX) and spatial sampling density.

**Results:**

The suitability of the imaging strategy is evaluated by comparing MV segmentations against ground truth annotations. The generated synthetic images were compared to ones acquired with similar parameters, and the result is promising. The quantitative analysis of annotation results suggests that the rLAX sampling strategy is preferable for MV assessment, reaching accuracy values that are comparable to or even outperform literature values.

**Conclusion:**

The proposed approach provides a valuable tool for the evaluation and optimization of cardiac valve image acquisition. Its application to the use case identifies the radial image sampling strategy as the most suitable for MV assessment through MRI.

**Supplementary Information:**

The online version contains supplementary material available at 10.1007/s11548-023-03012-y.

## Introduction

In biomedical engineering, the term phantom refers to an artificial object that has relevant properties of the human body or a medical device and can be used for multiple biomedical applications [1]. Phantoms play a significant role in the development of cardiac imaging and image analysis methods.

Physical phantoms are used to assess imaging device performance, geometric distortion, signal-to-noise ratio (SNR), etc., and provide important information for the development and validation of imaging and post-processing pipelines; few physical phantoms support the simulation of motion and blood flow [2–6] see Table 1 in Supplementary material. Most published physical phantoms focus on sub-parts of the cardiovascular anatomy, such as the ventricles [3–5]. Few physical phantoms represent cardiac and breathing motion (https://www.cirsinc.com/products/radiation-therapy/dynamic-cardiac-phantom/ retrieved on 14.07.2023). In addition to physical phantom models, numerical phantom methods are widely used in the development of imaging techniques for moving structures such as the heart. These are more easily adaptable for changes in the anatomy-pathology of the relevant structures, see Table 2 in Supplementary material. For image acquisition optimization purposes, the combination of computational human phantoms with the simulation of the imaging procedure can be a time- and cost-efficient tool [7–17].

**Table 1:** Properties of the datasets used for model generation. Thoracic anatomy and heart model were based on two CT datasets covering the torso of one patient as well as three CT datasets showing the heart anatomy of different patients. MRI intensity properties were extracted from case 4. CT volumes 1.2, 2, 3 consist of ten phases, from which we choose the timeframe with open MV

Case/volume	Image size	Voxel size [mm^3^]	Data type	Model components
1.1	512 × 512x345	0.65 × 0.65 × 2	CT	Thorax
1.2	256 × 256x286	0.86 × 0.86 × 0.5	CT	Aorta, atria, ventricles and MV
2	256 × 256x300	0.68 × 0.68 × 0.5	CT	
3	512 × 512x240	0.36 × 0.36 × 1	CT	
4	320 × 320x12	0.87 × 0.87 × 6	MRI	Intensities

**Table 2:** Intensity distributions. Intensity distributions mean and standard deviation (SD) values for each entity in the anatomical 4D model extracted from exemplary CMR (case 4 in Table 1)

		Intensity mean	Intensity sd
Left heart	Mitral valve	266	25
	Atrial wall	100	38
	Atrial cavity	357	14
	Myocardium	75	8
	Ventricle cavity	344	23
Right heart	Atrial wall	88	32
	Atrial cavity	405	26
	Ventricle wall	71	25
	Ventricle cavity	356	15
	Aorta	330	22
	Bones	157	72
	Liver	119	47
	Kidney	126	14
	Lung	8	4
	Bronchi	389	42

The development of appropriate imaging strategies that enable a comprehensive assessment of cardiac anatomy, morphology, and function requires the consideration of the capabilities of the imaging system as well as the cardiovascular motion induced by contraction and breathing.

Computational phantoms for the simulation of cardiac image data consist of three major modules,

- 1.Generation of the anatomical model,
- 2.Simulation of the motion, and
- 3.Simulation of the imaging process/properties of image data.

### Anatomy

Existing geometrical models of selected cardiac structures are based on a mathematical ellipsoidal model [17] or image segmentations [10, 13, 14, 16]. Advanced models such as the XCAT phantom have been extended over the years so that the anatomical model covers the thorax and provides a detailed heart model. The phantom can be parameterized to represent different genders and age groups [18].

### Motion simulation

Regarding motion simulation, observation-based as well as finite element simulations have been suggested. Models based on observations typically perform statistical analysis of respiratory and cardiac motion [10, 11]. The models presented by Zhou et al. [14] and Baillargeon et al. [12] model the electromechanical behavior of the heart to simulate cardiac contraction. Segars et al. proposed a model based on 4D nonuniform rational b-splines (NURBS) [7, 9] and in [15] further consider hemodynamics. These models enable simulations for different parameterizations in order to consider e.g., disease-related changes in the blood flow or excitation propagation [12].

### Imaging simulation

Similar to motion simulation, image generation can be based on observations or physics-based simulation models. Hanafy et al. used observations of image data in combination with a Poisson noise model for the simulation of cardiac SPECT images [17], and Gilbert et al. employed a CycleGAN to generate synthetic echocardiographic images based on machine learning [16]. The observation-based approaches are very successful in the generation of realistic-looking images, which consider the typical artifacts and properties of real images. However, they can only reproduce the properties of the observed training sets. The CT simulations provided by Segar et al. use the analytical projection algorithm to simulate image acquisition in order to support the optimization of data acquisition and processing [8, 10]. For MRI imaging simulations, Wissmann et al. applied a simulation operator, which considered tissue properties, imaging sequence, coil and noise [11], whereas Zhou et al. parameterized the OD1N simulator (http://od1n.sourceforge.net/ retrieved on 05.09.2022) with the literature parameters to generate synthetic cardiac MRI images [14].

To date, none of the above-mentioned methods supports the investigation of mitral valve imaging strategies. The mitral valve (MV) is composed of very thin tissue moving rapidly [19]. Each MV component can present structural abnormalities that can affect its function [20]. There are two computational cardiac phantoms, which include a model of the mitral valve; but they have so far only been used in the context of device design, and disease and treatment-related simulations [8, 13]. Furthermore, they do not consider the surrounding structures. Gao et al. proposed a model limited to mitral valve-ventricle coupling [13] and Baillargeon et al. present a four-chamber heart model [12].

Cardiac magnetic resonance (CMR) imaging has the potential for a comprehensive evaluation of the mitral valve. The quantitative assessment of the severity of MV dysfunction can support patient-specific therapy planning [21]. Lang et al. demonstrated that radially rotated long axis image sampling strategies have the potential for accurate mitral annulus identification [22]. Current MRI imaging protocols do however not include optimal sequences for the quantitative assessment of the complete mitral valve anatomy, including orifice and leaflets, and motion [23]; the identification of a suitable sampling strategy is therefore highly desirable. A computational phantom that includes a mitral valve could help identify the optimal imaging strategies to improve the assessment of mitral valve anatomy and motion properties.

The goal of our work is the development of a numerical simulation framework, which supports the investigation of imaging strategies for the mitral valve. This includes the identification of optimal spatio-temporal sampling to delineate the MV anatomy. We propose a computational phantom based on thorax CT and cardiac CT segmentations. Position-based dynamics simulate the closing dynamics of the MV [24]. We combine this dynamic model with a framework for the application of different intensity distribution models and sampling strategies. We test the applicability by evaluating different MRI image planes against segmentation and quantification accuracy.

## Materials and methods

This section describes the proposed computational phantom for synthetic 4D image generation and the use case evaluating different spatiotemporal sampling strategies for MRI imaging regarding MV annotation accuracy.

### Computational phantom

#### Data for model generation

The ground truth geometric model is generated with segmentations of contrast-enhanced CT datasets (Siemens SOMATOM Definition Flash, Siemens, Germany). Data from three adult patients suffering from type II insufficiency were selected. Informed consent was obtained and ethical permission was granted (EA2/093/16 or EA2/133/14). For our example use case, we processed four CT image volumes; a torso CT was used to extract anatomical representations of relevant thoracic structures such as ribcage, lung and aorta. The three cardiac CT angiography images for cases 1–3 were used for the more detailed heart and valve segmentations (details in Table 1).

For the extraction of image properties used in the simulation of the synthetic images, intensity values of the corresponding anatomical structures and their standard deviation were extracted from in vivo cardiac cine MRI images (case 4 in Table 1) [25].

#### Anatomical model with a moving mitral valve

The image-based anatomical model is generated by combining relevant entities:

- Relevant anatomical structures (bones, lung and bronchi, liver, kidney, aorta) in the **torso** were segmented interactively in the first dataset of case 1 (Table 1) using an ad hoc tool developed in MeVisLab [26].
- The **heart** (composed of both atria and ventricles) was segmented with Brainview (Philips Healthcare, Best, the Netherlands) using the cardiac CT data for all three cases. These segmentation methods provide voxelmasks with spatio-temporal resolutions determined by the input image data.
- The diastolic phase with open MV was chosen from the multiphase cardiac CT images, and the segmentations were provided by an experienced user. The open **mitral valve** was segmented for all three cases with the method proposed by Tautz et al. [27]. This method provides a smooth surface mesh with a sampling that is independent of the resolution of the underlying image data. The mean face area of our mesh model of the valve is 0.55 mm^2^. The resulting surface mesh was used as input for a valve closure simulation [24] with a temporal resolution of 180 frames/valve closure. The valve closure segmentation can deal with a moving annulus. For this work, we consider a static heart model and a fixed valve annulus. The valve surface was then rasterized to generate a voxelized image representation considering an appropriate surface thickness of 1 mm [28].

**Fig. 1:**
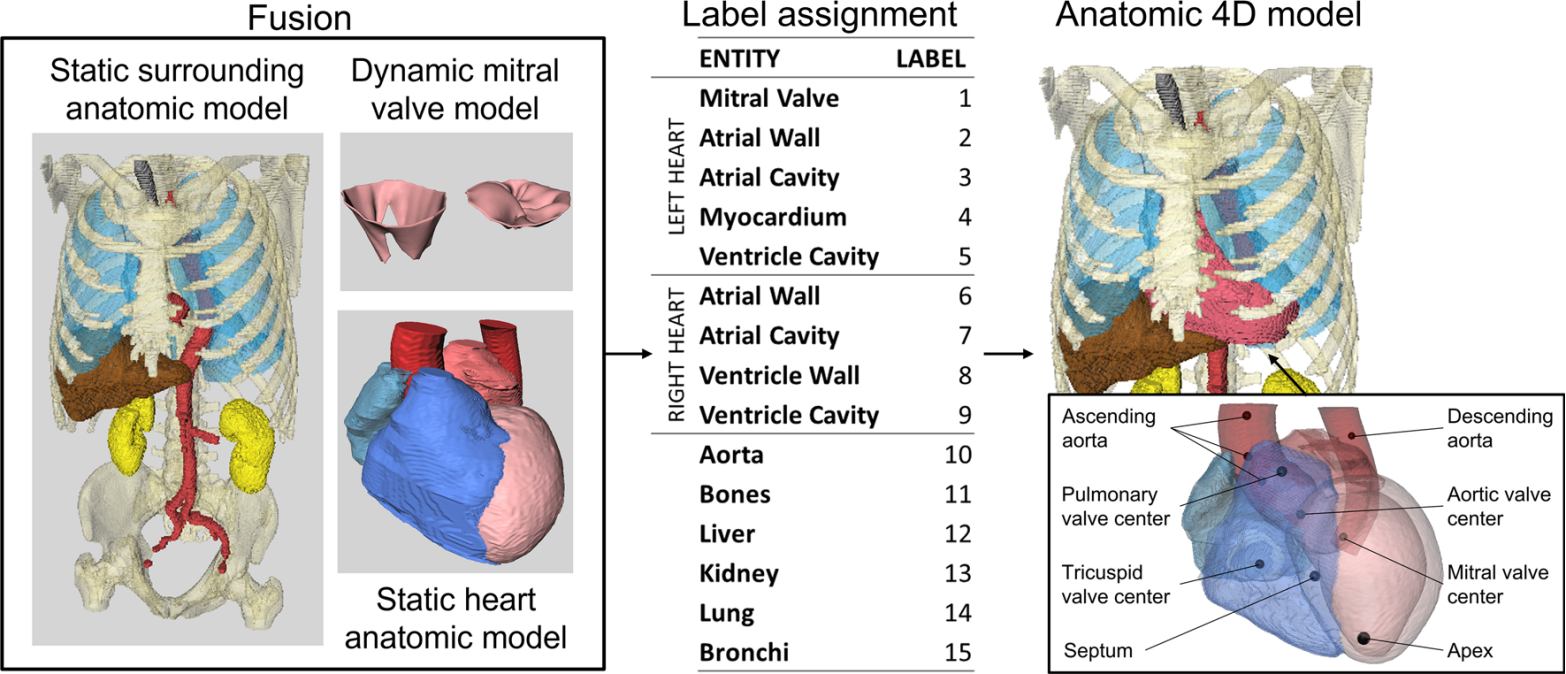
Model generation. The separate entities (left) are fused, and labels are assigned for all anatomical structures (right). The markers used for the fusion of the heart model with the surrounding structures are shown in the right box

To combine these components, the thorax segmentation model (from volume 1.1 in Table 1) was separately fused with each of the three heart segmentations (case 1.2–3 in Table 1) using 10 anatomical landmarks defining cardiac structures, including the four valves center, ventricle landmarks such as the apex and the center of the septum, and markers along the aorta centerline (Fig. 1). Corresponding landmarks were manually annotated in the thorax and the cardiac CTs, the distance between each marker pair was minimized by the iterative closest point algorithm [29] to obtain the complete model. The resulting geometric model for volume 1 (image size 512 × 512 × 345 and voxel size 0.65 × 0.65 × 2 mm^3^) is shown in Fig. 1.

#### Synthetic image generation

**Fig. 2:**
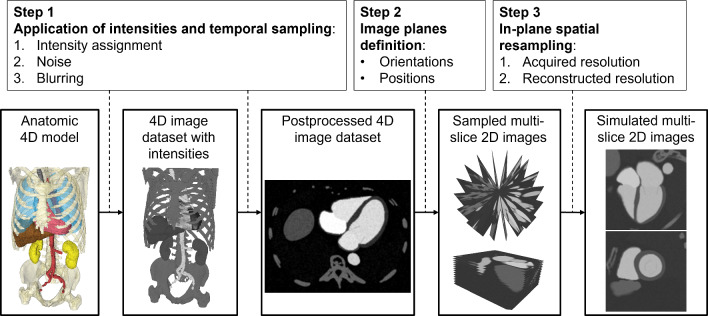
Image simulation workflow. Workflow for multi-slice 2D images simulation starting from the anatomic 4D model

The synthetic images are generated combining the 4D anatomic model and the observation-based intensity model following the workflow shown in Fig. 2, which consists of three major steps:

1. Application of intensity model and temporal sampling
2. Definition of position and orientation of image planes
3. In-plane spatial sampling

### Use case: MRI image simulation

#### Intensity model for the MRI imaging use case

**Fig. 3:**
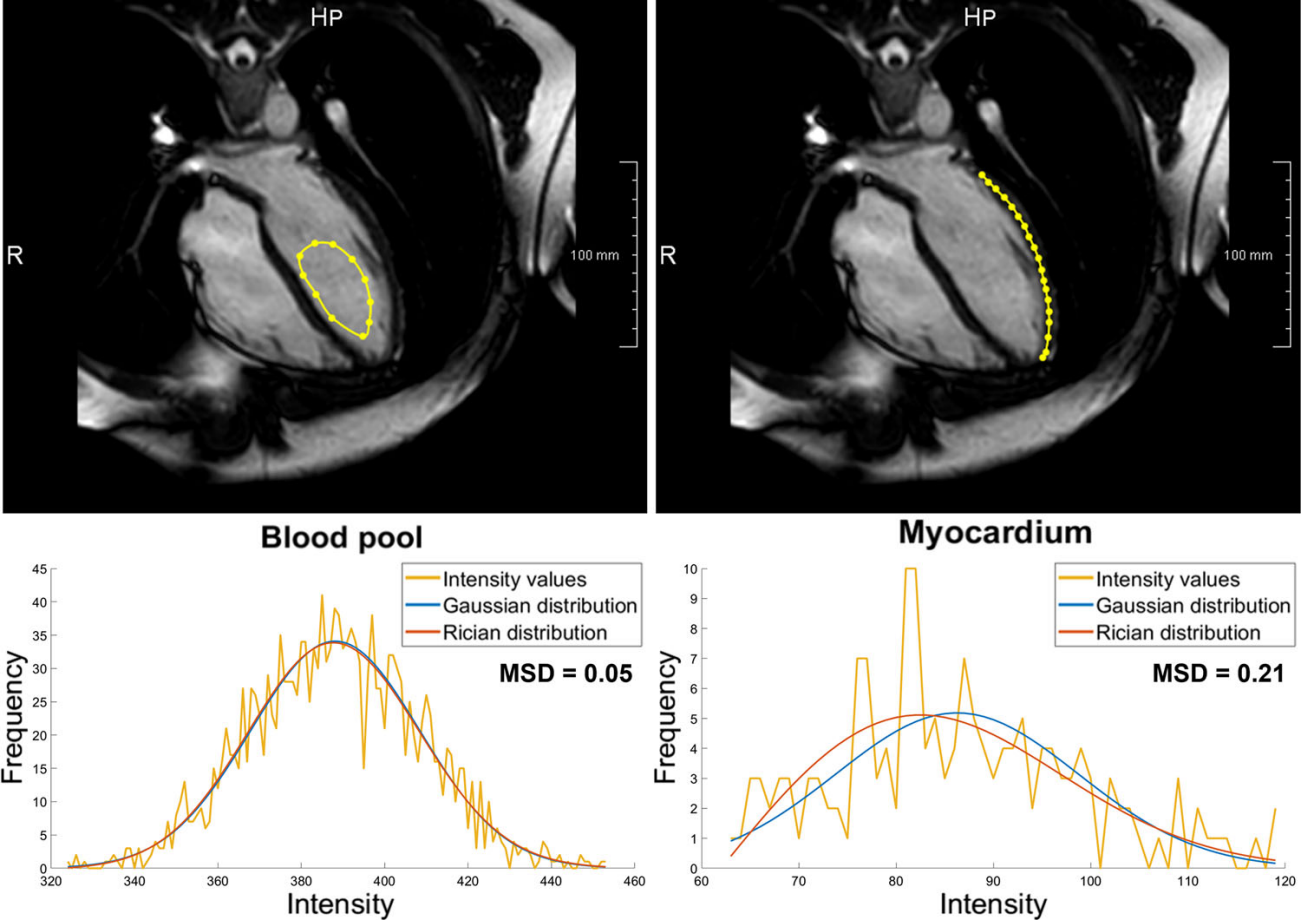
Segmentation for intensity distribution analysis. Examples of relevant representative regions (first row) for ventricle blood pool (left), and for the myocardium (right). The second row shows the corresponding intensity distributions (yellow) and the two fitted distribution models: Gaussian (blue) and Rician (orange). Mean square distance values (MSD) between the two curves are reported

The relevant entities such as lung, bones, blood pool, myocardium, etc. were segmented on a typical cardiac cine 4CH MRI dataset (case 4 in Table 1, full MRI properties in Table 3). The intensity distribution of each entity was analyzed with both Gaussian and Rician distribution fitting (Fig. 3) [30]. Given the minimal differences between the fitted distributions (mean square distances 0.05 and 0.21 for blood pool and myocardium, respectively), we decided to use the Gaussian for the simulation. Table 2 shows the corresponding mean and standard deviation for all intensity distributions.

**Table 3:** MR imaging parameters

Imaging Parameter	Values
Scanner	Philips ingenia ambition X 1.5 T
Series description	cine-4CH
Scanning sequence	Gradient recalled (GR)
Repetition time [ms]	3.3
Echo times [ms]	1.67
Flip angle [°]	60
In-plane resolution—acquired (isotropic) [mm]	1.4
In-plane resolution—reconstructed (isotropic) [mm]	0.866
Slice thickness [mm]	6
Slice spacing [mm]	6
Temporal resolution [phases/cycle]	30
K-space sampling	Cartesian

#### Application of intensities and temporal sampling

The observed intensities (Table 2) were assigned to each anatomical entity. In the next step, we added typical imaging artifacts such as noise and spatio-temporal partial volume effects (Fig. 2). First, temporal sampling was performed to obtain a resolution of 10 timesteps/valve closure, mimicking typical temporal resolutions observed in MRI images. Then, Gaussian noise (mean = 0 and σ = 38) was added. The value of σ was chosen as the maximum standard deviation within the values observed in the heart entities intensity distributions (left and right heart in Table 2). Lastly, smoothing with a Gauss kernel was applied, emulating the filtering applied in the reconstruction process [31].

#### Definition of position and orientation of image planes

The simulated image orientations were based on two standard views in cardiac MRI: short-axis (SAX) and radial long-axis rotation (rLAX). Automatic methods for SAX slice positioning described in literature use connection of the MV center and the LV apex as plane normal to determine the SAX orientation [32]. To emulate this approach, we determine the orientation of the SAX is based on the principal component analysis (PCA) of the MV to orient the image planes automatically parallel to the valve annulus. The minimum principal component (defining the valve axis) was used as the image plane normal. Both the gap between slices and slice thickness were set to 6 mm mimicking a typical short axis acquisition [25].

We defined three rotational long axis sampling schemes, consisting of 6, 9 and 18 slices (rLAX6, rLAX9, rLAX18). Image slices were created by rotating around the valve axis with a fixed angle. The slice thickness was set to 6 mm.

#### In-plane spatial sampling

Two consecutive spatial resampling steps were applied to mimic the acquired/reconstructed voxel size of MRI image data [25].

Spatial resolution values were set to those observed in the standard short axis images for both SAX and rLAX to allow comparison. Resampling to the acquired in-plane voxel size was performed first with a resolution of 1.4 × 1.4 mm^2^. The resampling to the reconstructed in-plane resolution was then applied using the observed resolution of 0.87 × 0.87 mm^2^.

#### Assessment of mitral valve annotation accuracy for different sampling strategies

The anatomic 4D model (Fig. 2) provides us with a ground truth anatomy for our segmentation analysis. This cannot be generated from MV measurements on acquired images, because only measured image values are available there and no reference method for quantitative valve assessment in vivo is available. All the comparison with ground truth performed in this session refer to the valve model with the simulated motion used as input for the synthetic image generation.

#### Mitral valve annotation

**Fig. 4:**
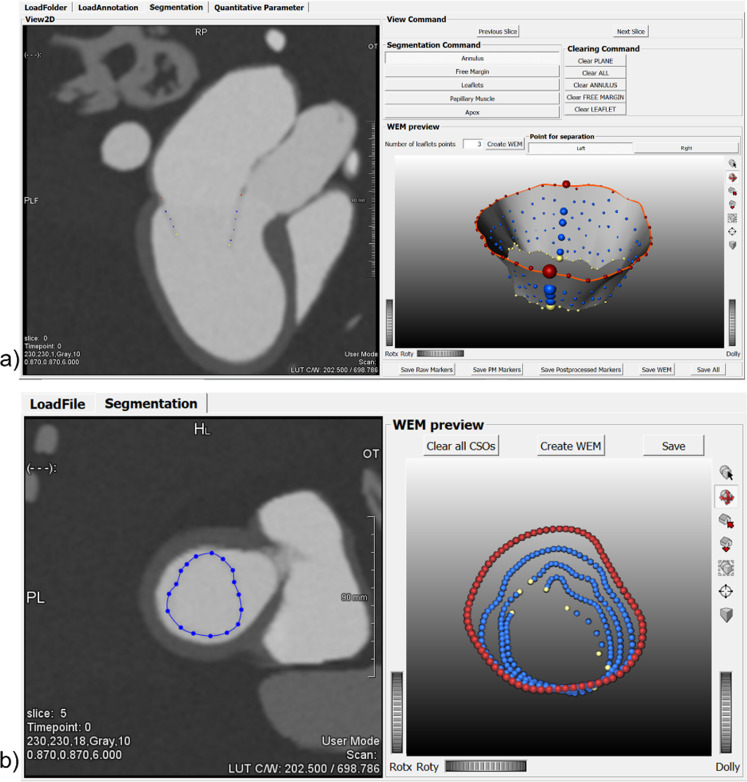
Annotation tools. Annotation software interface for the interactive annotation of the radial long axis (rLAX) images (**a**) and short axis (SAX) image data (**b**). Red points indicate the annulus, blue ones the leaflets and the yellow ones the leaflets end (orifice)

Three domain experts segmented the mitral valve in the simulated images using a dedicated prototypical software solution as shown in Fig. 4 (average segmentation time approximately 20 min per case; SAX 3.5 min, rLAX6 3.5 min, rLAX9 5 min and rLAX18 8 min); the valve annulus is highlighted with red points and the orange contour. The yellow points represent the valve orifice. The users annotated two points for the annulus, two for the leaflet ends (defining the orifice) and a variable number of points for leaflet contours on each rotational plane (rLAX). In the SAX stack the valve annotation was performed using spline contours.

#### Quantitative parameter calculation

For the quantitative assessment of the annotations, clinically established quantitative parameters [33] were computed for the segmented geometric valve model and the ground truth model.

**Fig. 5:**
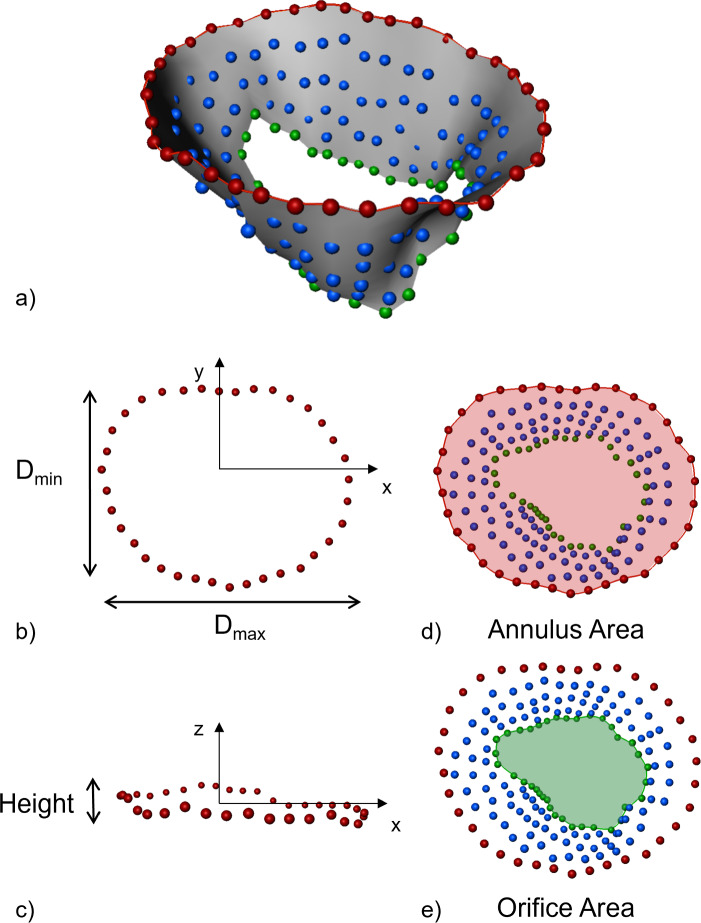
Clinically established quantitative parameters. Mitral valve model output from annotations (**a**) and clinically established quantitative parameters: Dmax: maximum diameter (**b**), Dmin: minimum diameter (**b**), height (**c**), annulus 2D area (**d**) and orifice 2D area (**e**). The valve axis used to set the plane orientation corresponds to the z-axis of the annulus PCA (**c**)

Annulus diameters and height were computed using a principal component analysis (PCA) on the annular points. Annulus and orifice areas were computed as two-dimensional areas after projecting the points to the annular plane (Fig. 5).

The values computed on the three expert annotations are compared for each case to its ground truth value.

Biases were computed for each quantitative parameter of each case for all the proposed sampling strategies as:

$$ BIAS = \frac{{\mathop \sum \nolimits_{i = 1}^{N} \left( {a_{CASE,i} - a_{GT} } \right)}}{N} $$

where ${a}_{CASE,i}$ is the value of the quantitative parameter computed on the user $i$ annotation of the analyzed case, ${a}_{GT}$ is the same parameter for the ground truth valve and $N$=3 is the number of users that performed the annotations.

#### Annulus and orifice contour

The shortest distances between the user segmented valve contour points and the relative ground truth contour were computed. Boxplots are used to report the results including minimum values, percentiles (25th, 50th and 75th), mean, maximum and outlier values. The corresponding mean and standard deviation values are reported in Supplementary material.

#### Point to surface distance

The shortest distance of each annotated point from the ground truth valve surface was computed. Color-coded glyphs were created for visualization, mean and standard deviation values were computed for each case and each user segmentation.

## Experiments and results

### Synthetic image generation

We compared the simulated image data to volunteer data acquired with similar parameter settings as suggested for rLAX18 (Table 3).

**Fig. 6:**
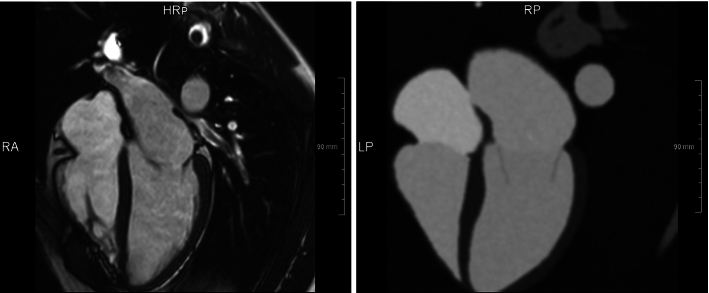
Real vs synthetic images comparison. MRI acquired on healthy volunteer (left) with similar parameters to our rLAX18 generated synthetic image based on the anatomy derived from CT and intensities from CMR (right)

Figure 6 shows corresponding slice orientations. It can be observed that intensity distributions appear similar. However, typical artifacts from hemodynamics as well as small structures not included in our anatomical model are missing in the simulated data, and anatomical regions appear more homogenous.

#### Annotation accuracy assessment

Three domain experts (a cardiologist, a cardiovascular surgeon, and a biomedical engineer, all experienced in MV annotation on echocardiographic data) segmented the mitral valve on the generated image datasets with the MeVisLab-based prototype (Fig. 4). On rLAX slices, the users annotated two points per slice to indicate the annulus position, two for the leaflet end and a variable number of points for the leaflet's contour. On SAX slices, the user annotated the valve using splines on each slice where the valve was visible.

The point clouds obtained from the segmentation on the simulated images with different sampling concepts were evaluated against ground truth annotations to quantify the achievable accuracy for valve assessment.

#### Quantitative parameter evaluation

**Fig. 7:**
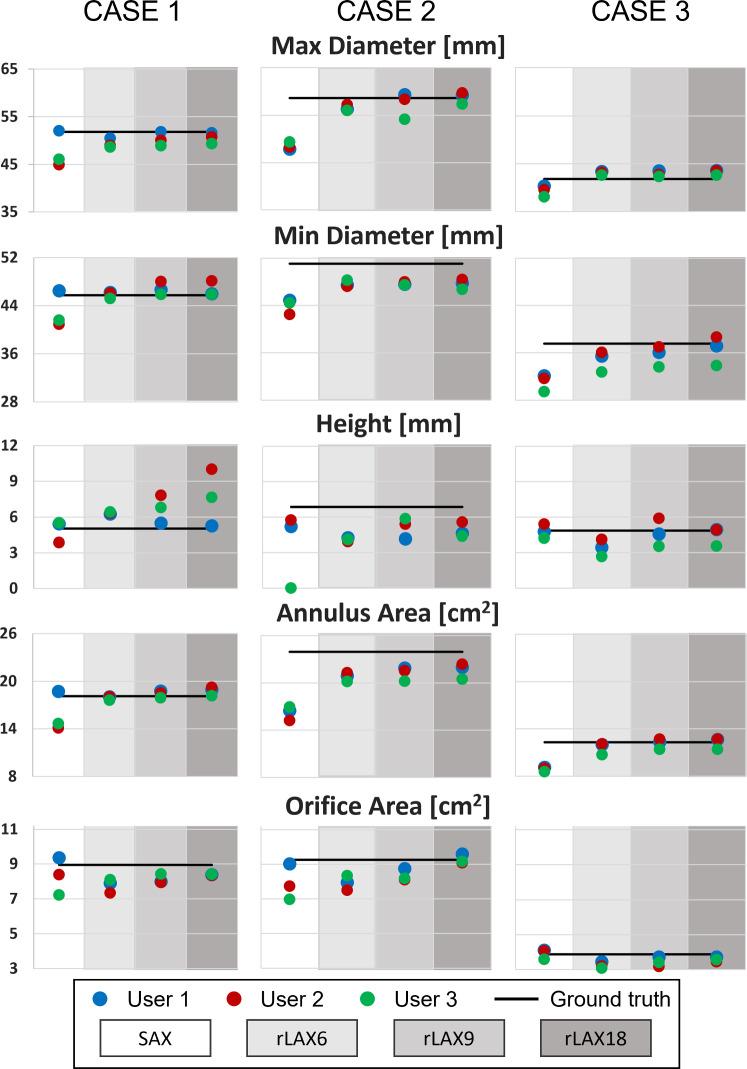
Quantitative parameters for case 1 (left), case 2 (middle) and case 3 (right) for all users (user 1 in blue, user 2 in red and user 3 in green). The black line corresponds to the value computed on the ground truth valve model. SAX: short axis, rLAX6, rLAX9, rLAX18: radial long axis with 6, 9 and 18 planes. The user segmentation values and relative distances from the ground truth are reported in Supplementary Material

The quantitative parameters computed for the anatomical models we generated based on the CT datasets and the PBD simulation are shown in Fig. 7. The inter-user variability is higher for SAX slices. On rLAX the differences between users decrease with the increasing number of rotational planes. Exceptions are observed for the height (case1 and case 3) and the minimum diameter (case 3). For most parameters, we observe the smallest errors for rLAX-derived ones.

Bias computed as shown in methods section are reported for each case and sampling strategies in Table 4. The values are in line with Fig. 7 finding and it can be observed a general underestimation of the valve in the user annotation on the synthetic MRI.

**Table 4:** Quantitative parameter bias for every sampling strategy (SAX, rLAX6, 9 and 18) and every case (case 1, 2 and 3). Minimum values for each quantitative parameter are in bold

BIAS [mm]					
	Quantitative parameter	SAX	rLAX6	rLAX9	rLAX18
CASE 1	Diameter max	−8.01	−4.70	−2.94	**−2.34**
	Diameter min	−6.08	**0.08**	2.36	1.93
	Height	**−2.28**	25.86	32.98	51.61
	Annulus area	−12.71	**−1.29**	1.38	3.53
	Orifice area	−7.10	−13.32	−9.39	**−6.38**
CASE 2	Diameter max	−17.30	−3.63	−2.30	**0.32**
	Diameter min	−13.99	−6.68	**−6.63**	−6.75
	Height	−46.79	−40.15	**−24.99**	−29.16
	Annulus area	−32.27	−13.16	−11.37	**−9.72**
	Orifice area	−14.82	−14.68	−10.02	**0.28**
CASE 3	Diameter max	−6.03	2.90	**2.47**	3.40
	Diameter min	−17.27	−7.45	−5.29	**−2.62**
	Height	**−1.10**	−29.60	−3.84	−7.71
	Annulus area	−27.63	−5.63	−1.19	**−0.50**
	Orifice area	**1.35**	−16.98	−11.91	−7.96

### Qualitative annulus and orifice contour evaluation

**Fig. 8:**
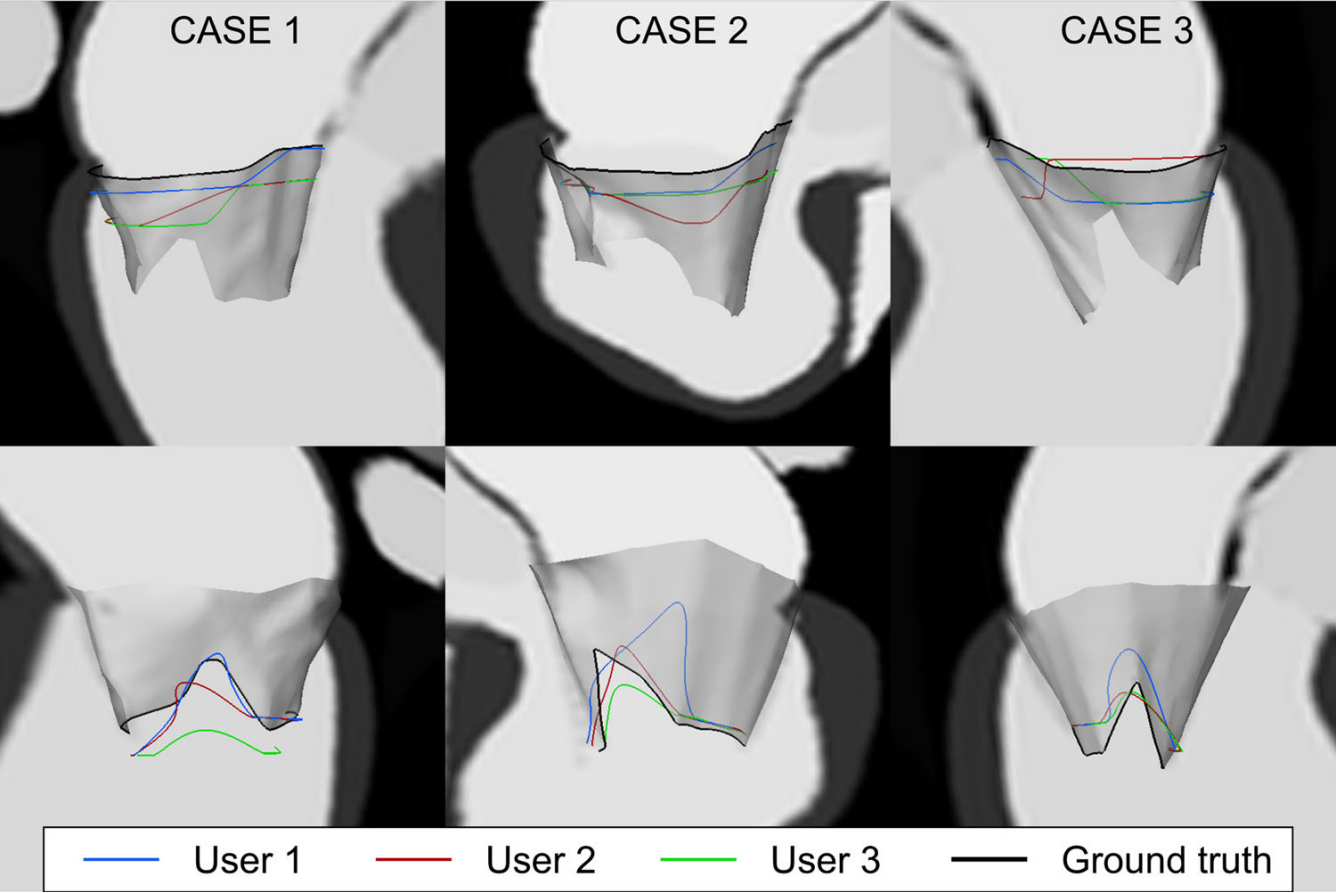
Annulus and orifice SAX annotations contours. Annulus (top row) and orifice profiles (bottom row) extracted from SAX annotations of all users (blue, red, green) and ground truth profile (black) for case 1 (left), case 2 (middle) and case 3 (right)

**Fig. 9:**
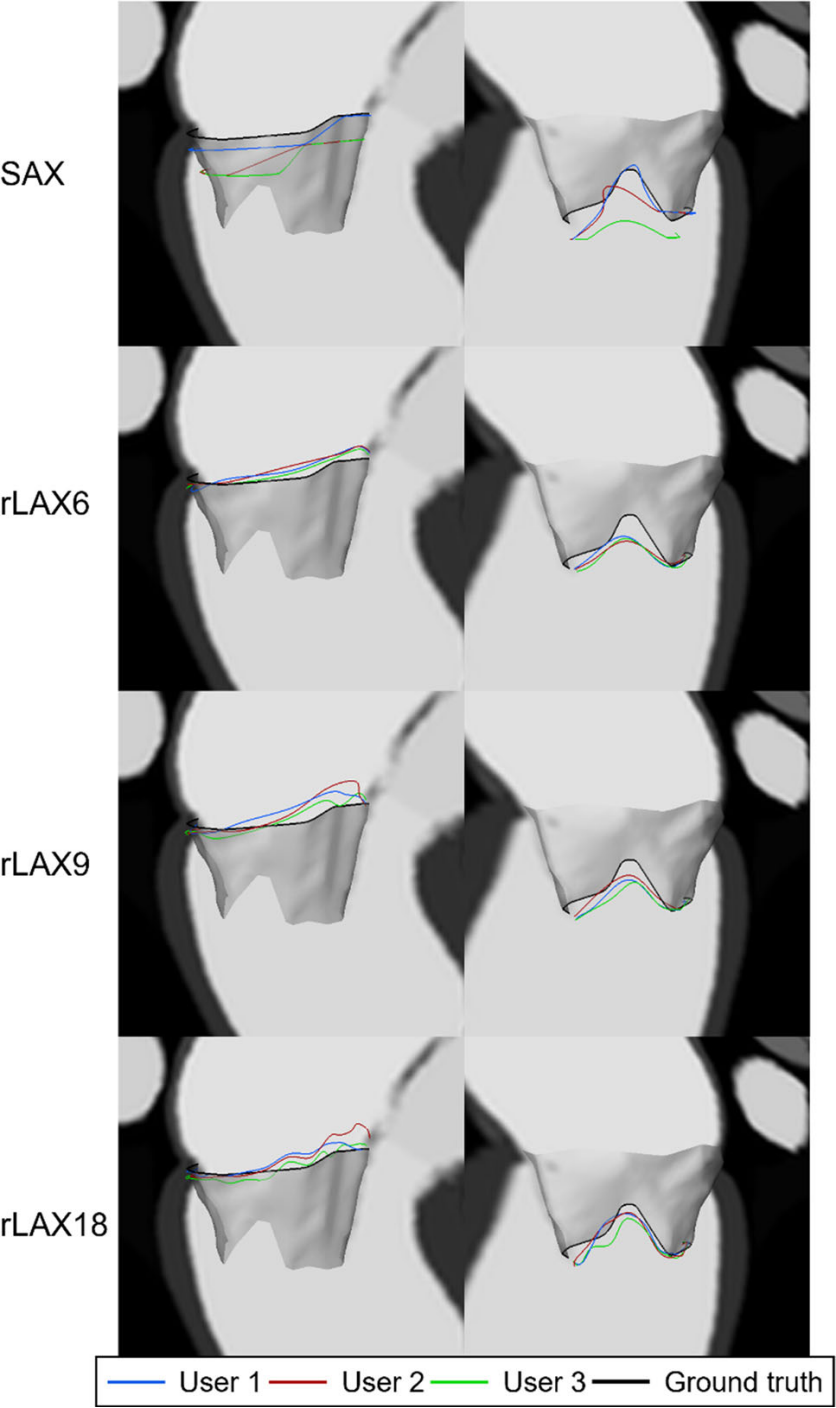
Annulus (left) and orifice (right) contours from the annotations of all users for case 1. From first row: short axis annotation (SAX), radial long axis with 6, 9 and 18 planes (rLAX6, rLAX9 and rLAX18). The best agreement for the orifice contour is achieved in the annotation of rLAX18

To understand the causes for the differences in the quantitative results, we qualitatively analyzed the annulus and orifice contours. Figure 8 shows the annulus and orifice contours obtained from the user annotations of the SAX images together with a surface visualization of the valve model. In all cases, the SAX-based contours differ most from the ground truth model. This could be due to sampling factors, such as slice spacing and thickness. The orifice contours do not match the ground truth, even when the orifice quantitative parameters seem to be well approximated also using SAX annotation (case 3 Fig. 7). Both contours were better approximated using rLAX annotations and their agreement with the ground truth improved increasing the number of planes (Fig. 9). The inter-observer agreement was also better for the rLAX approach.

### Annulus and orifice contour distance from ground truth

**Fig. 10:**
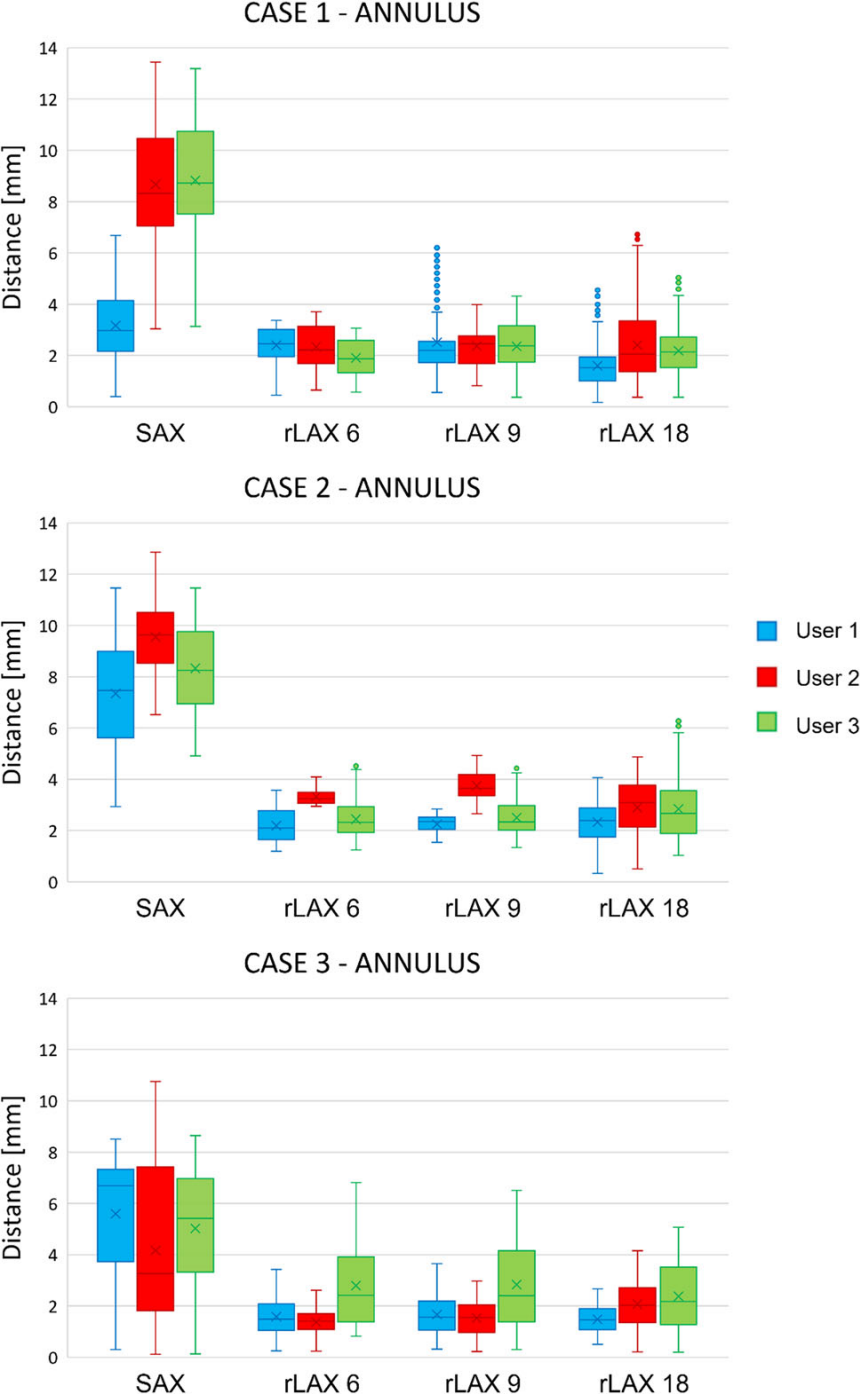
Annulus contour distances from the ground truth. Distances values between the ground truth annulus contour and the user annotations on the simulated images with different sampling strategies (SAX, rLAX6, rLAX9 and rLAX18). Mean and standard deviation are reported in Table 3

The annotation accuracy for annulus and orifice is assessed quantitatively via the distances between the ground truth and user contours. Figure 10 shows the distances between annotated annulus and the ground truth. The distances for SAX are higher than those from the rLAX annotations. Reference mean and standard deviation values are reported in Table 5.

**Table 5:** Annulus contour distance from ground truth. Average ± standard deviation in mm of the annotated annulus contour distance from the ground truth one. The minimum value for each user is highlighted in bold. SAX: short axis, rLAX: radial long axis with 6, 9 or 18 planes

		SAX	rLAX 6	rLAX 9	rLAX 18
CASE 1	User 1	3.16 ± 2.87	1.85 ± 0.92	2.10 ± 1.18	**1.60 ± 0.91**
	User 2	8.67 ± 2.40	**1.89 ± 1.00**	2.29 ± 1.51	2.30 ± 1.44
	User 3	8.83 ± 2.29	**1.74 ± 0.69**	2.14 ± 0.94	2.82 ± 0.89
CASE 2	User 1	7.34 ± 2.16	2.30 ± 0.94	**2.16 ± 0.81**	2.46 ± 0.96
	User 2	9.55 ± 1.49	3.09 ± 1.18	3.05 ± 1.28	**2.69 ± 1.32**
	User 3	8.33 ± 1.67	**2.80 ± 1.43**	3.13 ± 1.57	2.82 ± 1.19
CASE 3	User 1	5.60 ± 2.28	1.63 ± 0.62	1.69 ± 0.80	**1.48 ± 0.55**
	User 2	4.16 ± 2.98	**1.95 ± 0.92**	2.10 ± 0.83	2.07 ± 0.90
	User 3	5.03 ± 2.24	2.59 ± 1.36	2.52 ± 1.61	**2.38 ± 1.32**

**Fig. 11:**
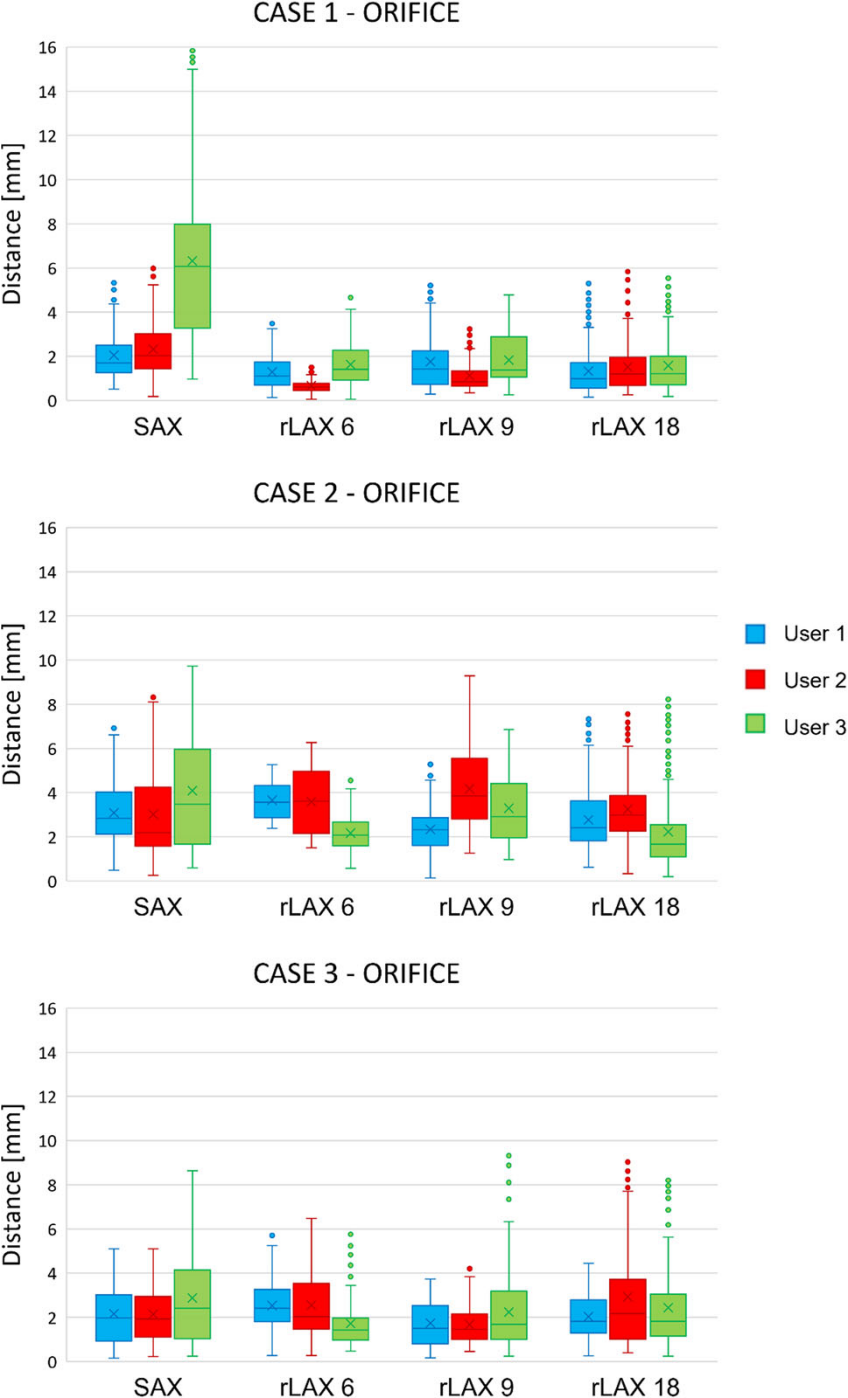
Orifice contour distances from the ground truth. Distances values between the ground truth orifice contour and the user annotations of the image data with different sampling strategies (SAX, rLAX6, rLAX9 and rLAX18). Corresponding mean and standard deviation are reported in Table 4

The orifice contour annotations on SAX also differ more from the ground truth than the rLAX annotations, even if the variation is slightly less evident in case 2 and case 3 (Fig. 11). Reference mean and standard deviation values are reported in Table 6.

**Table 6:** Orifice contour distance from ground truth. Average ± standard deviation in mm of the annotated orifice contour distance from the ground truth one. The minimum value for each user is highlighted in bold. SAX: short axis, rLAX: radial long axis with 6, 9 or 18 planes

		SAX	rLAX 6	rLAX 9	rLAX 18
CASE 1	User 1	2.05 ± 1.06	1.94 ± 1.62	1.79 ± 1.28	**1.33 ± 1.08**
	User 2	2.33 ± 1.32	2.35 ± 1.92	1.59 ± 1.11	**1.51 ± 1.16**
	User 3	6.32 ± 3.64	2.07 ± 1.68	1.95 ± 1.16	**1.58 ± 1.18**
CASE 2	User 1	3.09 ± 1.38	2.53 ± 1.27	**2.42 ± 1.26**	3.07 ± 1.60
	User 2	3.02 ± 1.92	4.22 ± 2.78	3.43 ± 1.73	**2.76 ± 1.59**
	User 3	4.09 ± 2.71	**1.85 ± 0.97**	2.45 ± 1.66	2.23 ± 1.76
CASE 3	User 1	2.17 ± 1.37	2.49 ± 1.38	**1.85 ± 0.95**	2.03 ± 0.98
	User 2	**2.14 ± 1.25**	2.52 ± 1.97	2.21 ± 1.51	2.93 ± 2.39
	User 3	2.88 ± 2.19	3.01 ± 2.95	2.66 ± 2.19	**2.44 ± 1.91**

### Distances of annotations from the ground truth surface

**Fig. 12:**
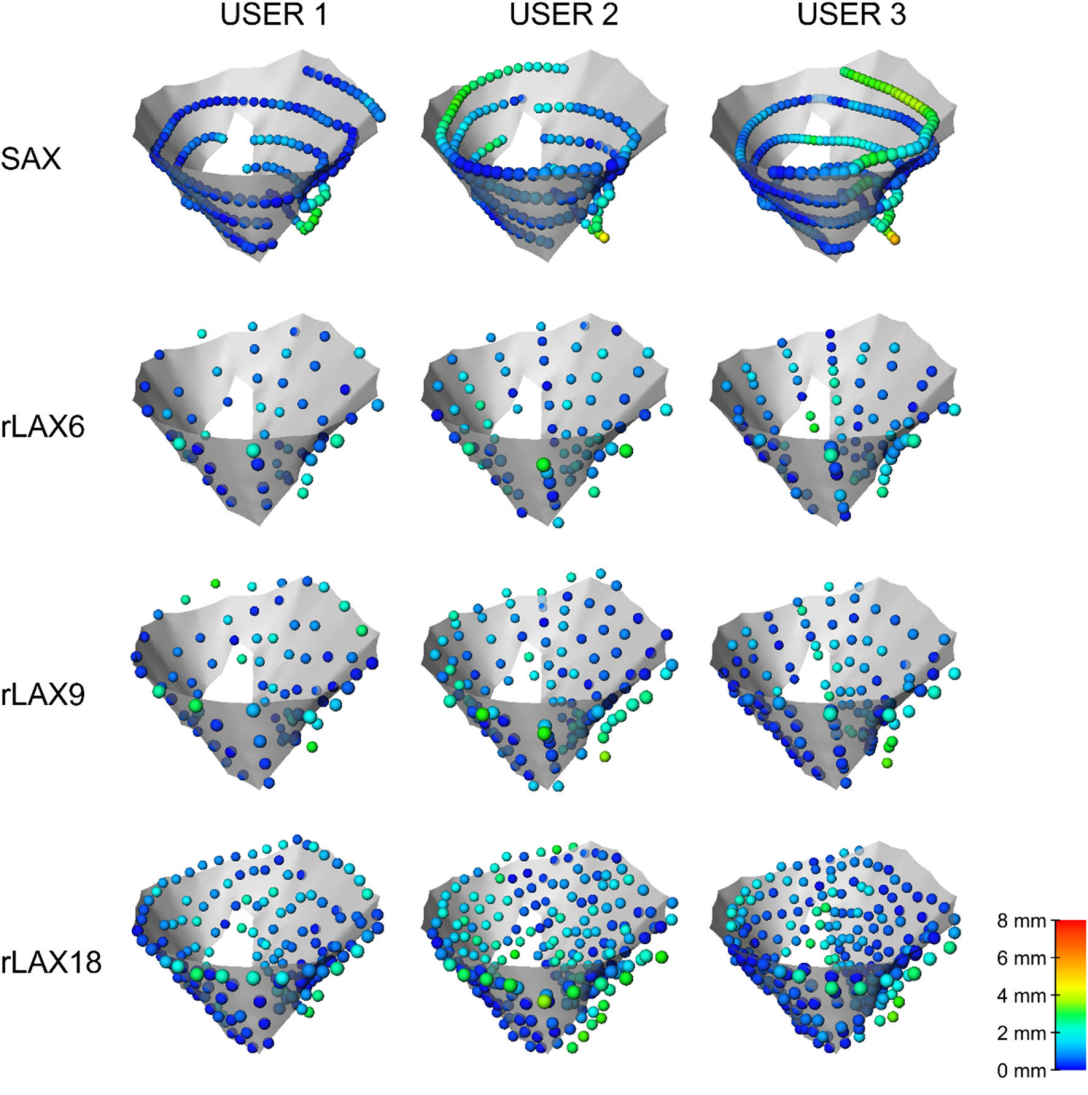
Distances of user annotations from ground truth valve surface for CASE 3. The points are color-coded depending on the distance from the surface, the scale is set according to the minimum and maximum distance values found for all three cases (see Supplementary Material)

The color-coded glyphs in Fig. 12 show positions and distances of the point annotations relative to the ground truth surface. The color scale is set according to the minimum and maximum distance values found in all three cases (see Supplementary Material).

It could be noted that precise annular shape information could not be derived from the SAX stacks. In addition, the points separating the leaflets and the leaflets tips were not correctly identified.

The average distances of the annotations from the ground truth surfaces are reported in Table 7. Even if the SAX image annotation points seem to be the closest to the ground truth surface for case 2, the analysis shown in Figs. 9, 10 and 11 demonstrates that the orifice and annulus contours differ substantially from the ground truth. We can observe that in some case SAX annotation allows correct identification of the visible parts of the valve but essential information about annulus and orifice is missing in the image data.

**Table 7:** Point-to-mesh distances. Average ± standard deviation of the computed point-to-mesh distances. All values are in mm and the minimum value for each user is highlighted in bold. SAX: short axis, rLAX: radial long axis with 6, 9 or 18 planes. Corresponding boxplots are reported in Supplementary material

		SAX	rLAX 6	rLAX 9	rLAX 18
CASE 1	User 1	1.10 ± 0.92	0.86 ± 0.67	0.93 ± 0.97	**0.79 ± 0.80**
	User 2	0.95 ± 0.82	0.87 ± 0.77	0.99 ± 1.11	**0.86 ± 1.07**
	User 3	1.39 ± 1.65	0.77 ± 0.58	0.77 ± 0.64	**0.69 ± 0.63**
CASE 2	User 1	**0.90 ± 0.87**	1.32 ± 0.88	1.15 ± 0.91	1.29 ± 1.00
	User 2	**1.15 ± 0.73**	1.77 ± 1.50	1.65 ± 1.22	1.50 ± 1.21
	User 3	**0.79 ± 0.66**	1.30 ± 0.91	1.37 ± 1.03	1.47 ± 1.00
CASE 3	User 1	**0.66 ± 0.62**	0.87 ± 0.63	0.95 ± 0.78	0.91 ± 0.64
	User 2	1.19 ± 0.92	1.17 ± 0.69	**1.10 ± 0.79**	1.20 ± 0.88
	User 3	1.21 ± 1.17	1.01 ± 0.68	0.92 ± 0.74	**0.85 ± 0.69**

### Application on healthy volunteer acquired MRI

**Fig. 13:**
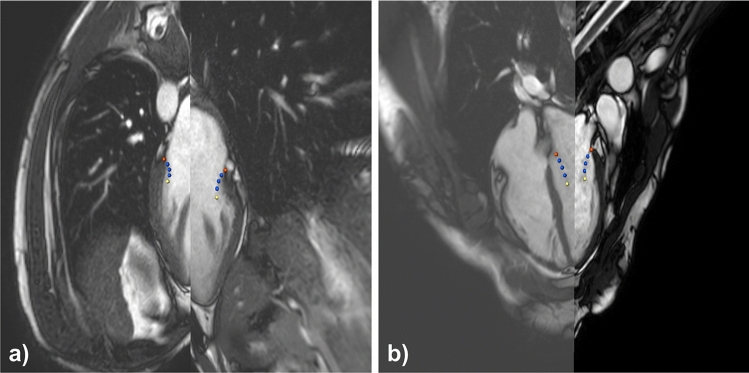
Valve positions in acquired images. Two different radial LAX images are shown in (**a**) and (**b**). Example (**a**) depicts a visible shift between two rotations. Example (**b**) shows a proper image acquisition

The annotation approach was tested on images acquired with the suggested sampling concept, the worst and the best results are shown in Fig. 13. The application highlighted the need for registration to correct for breathing motion.

**Fig. 14:**
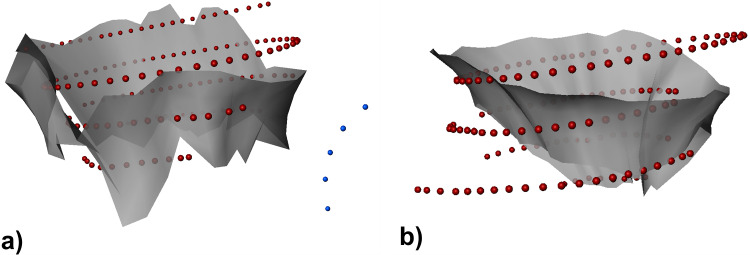
Image annotation results. Reconstructed valves from points segmented on rLAX are shown as gray surfaces. Annotations on SAX are shown as red points. The annotations on the completely misaligned rLAX plane are shown in blue and they have been excluded for the surface generation in (**a**). The surface borders corresponding to the annulus and the orifice are irregular in the worst case (**a**) even after excluding the most shifted plane

Annotation results from SAX and rLAX for worst and best case on healthy volunteer MRI are shown in Fig. 14. In both SAX cases, the annulus contour is flattened, and the commissures differ from the annotation on radial LAX.

For example, Figs. 13a and 14a show an irregular contour, meaning small shifts between slices in different rotations result in an implausible valve model.

## Discussion

The image data simulated with the presented approach look very similar to volunteer data acquired with similar spatio-temporal sampling, and the images have comparable properties regarding intensity distributions and noise. However, there are several features missing in the simulated data (Fig. 6). The effects of blood flow are not simulated, and ventricles appear more homogeneous than in real data. This observation corresponds to other simulation-based image generation approaches [11, 14]. Considering that the image generation approach by Gilbert et al. provides synthetic echocardiographic image data that looks realistic [16], a similar approach might also help to synthesize more realistic MRI images.

The valve annotation results provided for the different imaging strategies are directly connected to quantitative parameter estimation. Their variability affects information that are key in surgical planning, such as annulus shape and dimension, leaflet length (derived from orifice contour).

Quantitative parameters derived from rLAX annotations showed better agreement between users and with the ground truth reference values (Fig. 7), suggesting that this image sampling strategy would be preferable for clinically relevant parameters estimation. This result is in line with the analysis performed by Leng et al. [22] showing radial sampling strategies to be more accurate for annulus parameter identification. In the absence of the actual anatomy of the valve, the authors use values obtained on the 18 rotational long axis slices as ground truth. Standard methods suggested for the quantitative assessment of the mitral valve with MRI currently consider only 3 LAX orientations for mitral valve quantification [34]. Based on our observations, this might suffice for the detection of strong abnormalities, but not for the assessment of mitral valve properties in tasks which require the analysis of the valve leaflets e.g., for intervention planning.

Even though some quantitative parameters were derived correctly from the SAX stack annotation, the qualitative analysis of annulus and orifice contours shows that they were misplaced with respect to the ground truth.

The qualitative evaluation of the annulus and orifice contour (Figs. 8 and 9) further supports the idea that the anatomical coverage of the mitral valve on SAX does not allow for the extraction of the relevant landmarks with sufficient accuracy for an assessment of the individual anatomy.

The corresponding quantitative analysis confirms this hypothesis for both the annulus (Fig. 10) and the orifice contour (Fig. 11). Furthermore, the average differences for the annulus contour (rLAX6: 2.20 mm ± 1.01 mm, rLAX9: 2.35 mm ± 1.17 mm and rLAX18: 2.22 mm ± 1.05 mm) are substantially lower for rLAX than for SAX annotations (6.74 mm ± 12.12 mm). It is even lower than the error found in previous publications for mitral valve assessment (3.23 mm ± 2.66 mm) [27] and comparable to the lowest reported inter-user variation on echocardiographic data (1.63 mm ± 0.76 mm) [35]. The orifice contour differences follow the same pattern (rLAX6: 2.55 mm ± 1.84 mm, rLAX9: 2.26 mm ± 1.43 mm, rLAX18: 2.21 mm ± 1.52 mm, SAX: 3.12 mm ± 1.87 mm, literature [27]: 3.84 mm ± 2.54 mm).

The achievable annotation accuracy on SAX highly depends on the distance between the slices, their thickness and position. If the annulus is located between two slice planes, the annulus profile annotation will be misplaced (Fig. 8). rLAX images enable annotation of the leaflets from annulus to the end of the leaflets on every slice, but commissure identification is affected heavily by the number of considered rotations. Acquiring a higher number of slices increases the chance to intersect the valve in the commissure area but also means higher scanning and annotation times and effort. Our approach enables the optimization of the number of planes with regard to the required accuracy. Overall, our results indicated that an rLAX image sampling strategy would be preferable for MV assessment, reaching accuracy values that are comparable or even outperform literature values. This result is in line with the measure performed by Garg et al. [36] in which the authors recommend long-axis stack to best assess the MV leaflets.

Concerning the accuracy of our model with respect to other cardiac phantoms that include the mitral valve, the distances between the annotation on the synthetic images and the ground truth are comparable for all the cases and all the user to the segmentations reported by Laing et al. [37] for their physical patient-specific model (0.98 mm ± 0.91 mm).

We observed the need of registration in the MRI data acquired with the rLAX acquisition concept considered best according to the simulation study (Figs. 13, 14). Annulus and orifice could be better identified than on the SAX data, but an accurate valve model reconstruction would require a strategy to avoid or correct the motion induced shift between the rotations, which had been acquired in separate breathholds. Motion tracking is a common problem for cardiac valve imaging, and our approach could be extended with a motion simulation using parameters derived from real world data with MV tracking algorithms [38, 39]. Vice versa, our model could be helpful to validate automatic valve tracking algorithm since it provides ground truth reference values for accuracy analysis.

## Limitations

The MV apparatus model we used for the image generation was limited to the valve leaflets surface and annulus, although the simulation of the valve closure also considered the papillary muscles and the chords apparatus. Furthermore, the heart contraction is neglected in the image generation. Introducing the heart motion will also result in a moving annulus and valve and this could decrease the segmentation accuracy for the proposed sampling strategies.

The synthetic image generation considers only some of the imaging artifacts related to motion and image postprocessing. Artifacts caused by magnetic field imperfection [31] are not addressed and they should be included in future.

The limited number of 3 subjects used for development and validation of the model is a main limitation of the work and will be extended in future works.

## Conclusion

We presented a computational phantom for synthetic image generation that includes a simulation of the moving mitral valve. We presented its application for the evaluation of MRI valve imaging strategies. For the image-based assessment of the MV different image orientation and positioning strategies were evaluated, and the most promising strategy was applied for image acquisition in a healthy volunteer to test the transferability. The radial sampling strategy was found to be best for MV anatomy assessment. In line with [22], the results also suggest that rLAX6 and rLAX9 might be sufficient for the delineation of the annular profile. For the correct identification of the orifice, however, rLAX18 may be preferable.

General findings on the assessibility of key features of the mitral valve could be transferred from simulated to real image data. However, the dynamic model as well as the intensity simulation might benefit from the integration of a motion model and a machine learning approach for image synthesis as suggested in the related literature [10, 11, 16].

The presented use case investigates MRI imaging for MV assessment, the image synthesis pipeline can be applied to other imaging techniques as well as to other cardiac valves.

### Supplementary Information

Below is the link to the electronic supplementary material.

Supplementary file1 (DOCX 1783 KB)
