# Supplementary material for: A simulation-based phantom model for generating synthetic mitral valve image data–application to MRI acquisition planning
Source: Int J Comput Assist Radiol Surg. 2023 Sep 7;19(3):553–69. doi: 10.1007/s11548-023-03012-y (PMC10881710; doi:10.1007/s11548-023-03012-y)
Supplement: Supplementary file 1 — Supplementary file1 (DOCX 1783 KB) [file 11548_2023_3012_MOESM1_ESM.docx]

**A simulation-based phantom model for generating synthetic mitral valve image data – application to MRI acquisition planning**

Chiara Manini ^a, b^ (ORCID 0000-0001-5357-3396)

Olena Nemchyna ^c^ (ORCID [0000-0002-0564-533X](https://orcid.org/0000-0002-0564-533X))

Serdar Akansel ^c^ (ORCID [0000-0002-3479-4537](https://orcid.org/0000-0002-3479-4537))

Lars Walczak ^a, b, d^ (ORCID [0000-0001-6124-0671](https://orcid.org/0000-0001-6124-0671))

Lennart Tautz ^d^ (ORCID [0000-0002-1758-6111](https://orcid.org/0000-0002-1758-6111))

Christoph Kolbitsch ^e^ (ORCID [0000-0002-4355-8368](https://orcid.org/0000-0002-4355-8368))

Volkmar Falk ^c, b, f^ (ORCID [0000-0002-7911-8620](https://orcid.org/0000-0002-7911-8620))

Simon Sündermann ^c, b, f^ (ORCID 0000-0003-4927-1584)

Titus Kühne ^a, b, f^ (ORCID [0000-0003-1631-4824](https://orcid.org/0000-0003-1631-4824))

Jeanette Schulz-Menger ^b, f, g^ (ORCID 0000-0003-3100-1092 )

Anja Hennemuth ^a, b, d, f, h^ (ORCID [0000-0002-0737-7375](https://orcid.org/0000-0002-0737-7375))

- Deutsches Herzzentrum der Charité (DHZC), Institute of Computer-assisted Cardiovascular Medicine, Berlin, Germany
- Charité – Universitätsmedizin Berlin, corporate member of Freie Universität Berlin and Humboldt Universität zu Berlin, Berlin, Germany.
- Deutsches Herzzentrum der Charité (DHZC), Department of Cardiothoracic and Vascular Surgery, Berlin, Germany
- Fraunhofer MEVIS, Berlin, Germany
- Physikalisch-Technische Bundesanstalt (PTB), Braunschweig and Berlin, Germany
- DZHK (German Center for Cardiovascular Research), Partner Site Berlin, Berlin, Germany
- Helios Hospital Berlin-Buch, Department of Cardiology and Nephrology, Berlin, Germany
- Department of Diagnostic and Interventional Radiology and Nuclear Medicine, University Medical Center Hamburg-Eppendorf, Germany

**Corresponding author: Chiara Manini** [**chiara.manini@dhzc-charite.de**](mailto:chiara.manini@dhzc-charite.de)

- Supplementary Material
- *Physical heart phantoms for medical imaging*

***Table 1. Physical heart phantoms.***

|  | Paper | Year | Anatomical Model | Dynamics | Disease simulation | Personalizable | Modality | Applications |
| --- | --- | --- | --- | --- | --- | --- | --- | --- |
|  | D. Debrun et al. | 2005 | inner and outer wall of left myocardium | volume variation with an external pump | no | no | gated SPECT and 4D echo | Volume measurement |
|  | Y. Zhu et al. | 2014 | left and right ventricle | computer controlled ventricle motion | no | no | MRI | Validation of computational heart model |
|  | A. Krakovich et al. | 2021 | inner and outer wall of left myocardium | passive ventricle distension through a pulsatile pump | no | no | SPECT | Myocardial blood flow measurement validation on SPECT system |
|  | A. Krakovich et al. | 2023 | mechanical cardiac phantom [3] in anthropomorphic torso | passive ventricle distension through a pulsatile pump | no | no | SPECT | Myocardial blood flow measurement |
|  | [SUM NUCLEAR](https://charitede.sharepoint.com/sites/PhDProjectChiara/Shared%20Documents/General/Simulation%20of%20MRI%20Images/IJCARS%20review2/SUM%20NUCLEAR%20%20https:/www.cirsinc.com/products/radiation-therapy/dynamic-cardiac-phantom/)   [https://www.cirsinc.com/products/radiation-therapy/dynamic-cardiac-phantom/](https://charitede.sharepoint.com/sites/PhDProjectChiara/Shared%20Documents/General/Simulation%20of%20MRI%20Images/IJCARS%20review2/SUM%20NUCLEAR%20%20https:/www.cirsinc.com/products/radiation-therapy/dynamic-cardiac-phantom/) |  | whole heart | cardiac motion (translation and rotation) + breathing motion (motion control software) | no | no | CT | calcification detection, iodine contrast resolution and ECG signal gating |

- *Computational heart phantoms*

***Table 2. Computational heart phantoms.***

|  | Paper | Year | Anatomical Model | Dynamics | Disease simulation | Personalizable | Image Simulation | Modality | Applications |
| --- | --- | --- | --- | --- | --- | --- | --- | --- | --- |
|  | Segars et al. | 1999 | 4D NURBS | 4D NURBS observation based | no | no | no |  |  |
|  | Segars et al. | 2008 | model from Segars 1999 incorporated in torso | motion from Segars 1999 | no | no | analytical projection algorithm | CT |  |
|  | Segars et al. | 2009 | model from Segars 1999 incorporated in torso | motion from Segars 1999 | no | no | no |  |  |
|  | Segars et al. | 2010 | whole body model based on scaled segmentations | observation based respiratory and cardiac motion models | yes | no (XCAT) | analytical projection algorithm (Segars 2008) | CT | improve imaging instrumentation, data acquisition, techniques, and image reconstruction and processing methods |
|  | Wissmann et al. | 2014 | model from Segars 2010 | observation based respiratory and cardiac motion models | yes | no (XCAT) | simulation operators considering tissue properties, sequence, coil, noise | MRI | improve imaging instrumentation, data acquisition, techniques, and image reconstruction and processing methods |
|  | Baillargeon et al. | 2014 | Model based on CT and MRI image data | FE model with excitation-contraction coupling | yes | no | no |  | simulations for device design and treatment planning |
|  | Gao et al. | 2017 | MRI image-based LV-MV model with | FE-FSI model | no | yes | no |  | model-based advanced analysis of heart diseases |
|  | Zhou et al | 2018 | RV/LV image-based | electromechanical model | yes | yes | physical simulators (OD1N) with parameters for T1 and T2 from literature (<http://od1n.sourceforge.net/>) | Echo, MRI | evaluation of post-processing |
|  | Segars et al. | 2019 | extension of model from Segars 2010, finite element model of the whole heart based on Living Heart Project (Baillargeon et al.) | FE model with input of hemodynamic, electromechanical parameters | yes | no (XCAT) | analytical projection algorithm (Segars 2008) | CT | study the effects of anatomy and motion on medical images |
|  | Gilbert et al. | 2021 | Whole Heart Statistical shape model | none | no | yes | CycleGAN | Echo | synthetic data generation for training of AI models |
|  | Hanafy et al. | 2021 | mathematical ellipsoidal model | statistical volume models from observations | based on observations | no | observation-based with Poisson noise model | SPECT | evaluation of post-processing |

- *Quantitative parameter evaluation*

Quantitative parameter values computed on segmentation and corresponding ground truth ones are reported in Table 3, Table 4 and Table 5.

***Table 3. Quantitative parameter CASE 1.*** *Values computed on segmented valve and corresponding ground truth values.*

|  | **Quantitative parameter [mm]** | | | | | | |
| --- | --- | --- | --- | --- | --- | --- | --- |
|  | **Quantitative parameter** | **SAX** | **rLAX6** | **rLAX9** | **rLAX18** | **Ground truth** |  |
| **USER 1** | Diameter max | 51.99 | 50.44 | 51.82 | 51.63 | 51.78 |  |
|  | Diameter min | 46.49 | 46.19 | 46.66 | 45.98 | 45.79 |  |
|  | Height | 5.41 | 6.26 | 5.47 | 5.27 | 5.04 |  |
|  | Annulus Area | 1871.71 | 1802.77 | 1874.06 | 1893.50 | 1812.90 |  |
|  | Orifice Area | 916.35 | 770.86 | 778.80 | 819.24 | 874.23 |  |
| **USER 2** | Diameter max | 44.87 | 48.99 | 50.02 | 50.74 | 51.78 |  |
|  | Diameter min | 40.94 | 46.10 | 48.03 | 48.12 | 45.79 |  |
|  | Height | 3.85 | 6.34 | 7.83 | 10.01 | 5.04 |  |
|  | Annulus Area | 1410.37 | 1804.09 | 1850.78 | 1920.12 | 1812.90 |  |
|  | Orifice Area | 817.75 | 713.87 | 774.72 | 815.32 | 874.23 |  |
| **USER 3** | Diameter max | 46.03 | 48.61 | 48.92 | 49.34 | 51.78 |  |
|  | Diameter min | 41.58 | 45.18 | 45.90 | 45.90 | 45.79 |  |
|  | Height | 5.51 | 6.42 | 6.80 | 7.64 | 5.04 |  |
|  | Annulus Area | 1465.24 | 1761.66 | 1788.89 | 1817.29 | 1812.90 |  |
|  | Orifice Area | 702.39 | 788.69 | 823.01 | 820.91 | 874.23 |  |

***Table 4.*** ***Quantitative parameter CASE 2.*** *Values computed on segmented valve and corresponding ground truth values.*

|  | **Quantitative parameter [mm]** | | | | | | |
| --- | --- | --- | --- | --- | --- | --- | --- |
|  | **Quantitative parameter** | **SAX** | **rLAX6** | **rLAX9** | **rLAX18** | **Ground truth** |  |
| **USER 1** | Diameter max | 47.84 | 56.29 | 59.35 | 59.33 | 58.63 |  |
|  | Diameter min | 44.85 | 47.45 | 47.56 | 47.75 | 51.05 |  |
|  | Height | 5.19 | 4.25 | 4.16 | 4.61 | 6.84 |  |
|  | Annulus Area | 1646.26 | 2090.97 | 2187.00 | 2197.91 | 2393.10 |  |
|  | Orifice Area | 881.83 | 773.53 | 854.88 | 938.25 | 904.76 |  |
| **USER 2** | Diameter max | 48.28 | 57.20 | 58.39 | 59.74 | 58.63 |  |
|  | Diameter min | 42.46 | 47.23 | 47.96 | 48.38 | 51.05 |  |
|  | Height | 5.73 | 3.93 | 5.40 | 5.55 | 6.84 |  |
|  | Annulus Area | 1522.96 | 2126.18 | 2153.83 | 2236.89 | 2393.10 |  |
|  | Orifice Area | 753.78 | 728.60 | 789.55 | 888.60 | 904.76 |  |
| **USER 3** | Diameter max | 49.33 | 56.03 | 54.11 | 57.38 | 58.63 |  |
|  | Diameter min | 44.43 | 48.26 | 47.48 | 46.69 | 51.05 |  |
|  | Height | 0.00 | 4.10 | 5.84 | 4.38 | 6.84 |  |
|  | Annulus Area | 1693.34 | 2017.31 | 2022.10 | 2046.32 | 2393.10 |  |
|  | Orifice Area | 676.41 | 813.82 | 797.75 | 895.10 | 904.76 |  |

***Table 5.*** ***Quantitative parameter CASE 3.*** *Values computed on segmented valve and corresponding ground truth values.*

|  | **Quantitative parameter [mm]** | | | | | | |
| --- | --- | --- | --- | --- | --- | --- | --- |
|  | **Quantitative parameter** | **SAX** | **rLAX6** | **rLAX9** | **rLAX18** | **Ground truth** |  |
| **USER 1** | Diameter max | 40.32 | 43.35 | 43.48 | 43.55 | 41.80 |  |
|  | Diameter min | 32.10 | 35.42 | 36.07 | 37.16 | 37.53 |  |
|  | Height | 4.76 | 3.44 | 4.57 | 4.92 | 4.84 |  |
|  | Annulus Area | 912.62 | 1199.33 | 1236.67 | 1263.61 | 1230.42 |  |
|  | Orifice Area | 390.38 | 324.64 | 352.08 | 352.73 | 366.75 |  |
| **USER 2** | Diameter max | 39.50 | 43.10 | 42.74 | 43.51 | 41.80 |  |
|  | Diameter min | 31.66 | 36.08 | 36.99 | 38.65 | 37.53 |  |
|  | Height | 5.39 | 4.10 | 5.87 | 4.92 | 4.84 |  |
|  | Annulus Area | 900.96 | 1210.75 | 1268.34 | 1267.38 | 1230.42 |  |
|  | Orifice Area | 386.66 | 299.93 | 296.75 | 323.85 | 366.75 |  |
| **USER 3** | Diameter max | 38.02 | 42.59 | 42.29 | 42.60 | 41.80 |  |
|  | Diameter min | 29.38 | 32.71 | 33.57 | 33.83 | 37.53 |  |
|  | Height | 4.21 | 2.68 | 3.52 | 3.56 | 4.84 |  |
|  | Annulus Area | 857.65 | 1073.45 | 1142.44 | 1141.69 | 1230.42 |  |
|  | Orifice Area | 338.02 | 288.85 | 320.39 | 336.13 | 366.75 |  |

To better analyze the quantitative parameter computed, the relative difference percentage was computed using the Equation 1.

*Equation 1****.*** *Where a_CASE, i_ is the value of the parameter of the analyzed case for annotation from user i (i = 1, 2, 3) and a_GT_ is the same parameter of the ground truth case.*

The results are reported in Table 6, Table 7 and Table 8 for case 1, case 2 and case 3 respectively.

***Table 6*** ***Relative differences percentage CASE 1.*** *Relative differences percentage computed using Equation 1 on quantitative parameters from annotation points of each user for case 1. The minimum values found are highlighted in bold*

|  | **RELATIVE DIFFERENCES [%]** | | | | | | | |
| --- | --- | --- | --- | --- | --- | --- | --- | --- |
|  | **Quantitative parameter** | **SAX** | **rLAX6** | **rLAX9** | **rLAX18** | |  |  |
| **USER 1** | Diameter max | 0.41 | -2.58 | **0.08** | | -0.29 | |  |
|  | Diameter min | 1.53 | 0.89 | 1.91 | | **0.43** | |  |
|  | Height | 7.40 | 24.25 | 8.56 | | **4.54** | |  |
|  | Annulus Area | 3.24 | **-0.56** | 3.37 | | 4.45 | |  |
|  | Orifice Area | **4.82** | -11.82 | -10.92 | | -6.29 | |  |
| **USER 2** | Diameter max | -13.34 | -5.39 | -3.39 | | **-2.01** | |  |
|  | Diameter min | -10.58 | **0.68** | 4.90 | | 5.10 | |  |
|  | Height | **-23.63** | 25.82 | 55.41 | | 98.65 | |  |
|  | Annulus Area | -22.20 | **-0.49** | 2.09 | | 5.91 | |  |
|  | Orifice Area | **-6.46** | -18.34 | -11.38 | | -6.74 | |  |
| **USER 3** | Diameter max | -11.10 | -6.12 | -5.52 | | **-4.72** | |  |
|  | Diameter min | -9.19 | -1.32 | **0.257** | | 0.258 | |  |
|  | Height | **9.39** | 27.51 | 34.97 | | 51.63 | |  |
|  | Annulus Area | -19.18 | -2.83 | -1.32 | | **0.24** | |  |
|  | Orifice Area | -19.66 | -9.78 | **-5.86** | | -6.10 | |  |

***Table 7*** ***Relative differences percentage CASE 2.*** *Relative differences percentage computed using Equation 1 on quantitative parameters from annotation points of each user for case 2. The minimum values found are highlighted in bold*

|  | **RELATIVE DIFFERENCES [%]** | | | | | |
| --- | --- | --- | --- | --- | --- | --- |
|  | **Quantitative parameter** | **SAX** | **rLAX6** | **rLAX9** | **rLAX18** |  |
| **USER 1** | Diameter max | -18.40 | -4.00 | 1.22 | **1.19** |  |
|  | Diameter min | -12.15 | -7.06 | -6.84 | **-6.46** |  |
|  | Height | **-24.13** | -37.83 | -39.15 | -32.66 |  |
|  | Annulus Area | -31.21 | -12.62 | -8.61 | **-8.16** |  |
|  | Orifice Area | **-2.53** | -14.50 | -5.51 | 3.70 |  |
| **USER 2** | Diameter max | -17.65 | -2.45 | **-0.40** | 1.90 |  |
|  | Diameter min | -16.84 | -7.49 | -6.05 | **-5.23** |  |
|  | Height | **-16.24** | -42.53 | -21.14 | -18.87 |  |
|  | Annulus Area | -36.36 | -11.15 | -10.00 | **-6.53** |  |
|  | Orifice Area | -16.69 | -19.47 | -12.73 | **-1.79** |  |
| **USER 3** | Diameter max | -15.86 | -4.44 | -7.71 | **-2.13** |  |
|  | Diameter min | -12.98 | **-5.47** | -7.00 | -8.54 |  |
|  | Height | -100.00 | -40.08 | **-14.67** | -35.94 |  |
|  | Annulus Area | -29.24 | -15.70 | -15.50 | **-14.49** |  |
|  | Orifice Area | -25.24 | -10.05 | -11.83 | **-1.07** |  |

***Table 8*** ***Relative differences percentage CASE 3.*** *Relative differences percentage computed using Equation 1 on quantitative parameters from annotation points of each user for case 3. The minimum values found is highlighted in bold*

|  | **RELATIVE DIFFERENCES [%]** | | | | | | |
| --- | --- | --- | --- | --- | --- | --- | --- |
|  | | **Quantitative parameter** | **SAX** | **rLAX6** | **rLAX9** | **rLAX18** |  |
| **USER 1** | | Diameter max | -3.54 | **3.70** | 4.02 | 4.19 |  |
|  |  | Diameter min | -14.47 | -5.63 | -3.89 | **-0.99** |  |
|  |  | Height | **-1.61** | -28.86 | -5.52 | 1.71 |  |
|  |  | Annulus Area | -25.83 | -2.53 | **0.51** | 2.70 |  |
|  |  | Orifice Area | 6.44 | -11.48 | -4.00 | **-3.82** |  |
| **USER 2** | | Diameter max | -5.51 | 3.12 | **2.24** | 4.09 |  |
|  |  | Diameter min | -15.64 | -3.86 | **-1.43** | 2.99 |  |
|  |  | Height | 11.43 | -15.35 | 21.17 | **1.56** |  |
|  |  | Annulus Area | -26.78 | **-1.60** | 3.08 | 3.00 |  |
|  |  | Orifice Area | **5.43** | -18.22 | -19.09 | -11.70 |  |
| **USER 3** | | Diameter max | -9.05 | 1.88 | **1.16** | 1.92 |  |
|  |  | Diameter min | -21.71 | -12.85 | -10.54 | **-9.85** |  |
|  |  | Height | **-13.12** | -44.60 | -27.19 | -26.40 |  |
|  |  | Annulus Area | -30.30 | -12.76 | **-7.15** | -7.21 |  |
|  |  | Orifice Area | **-7.83** | -21.24 | -12.64 | -8.35 |  |

The minimum differences are obtained mainly on rLAX annotation. Different results were found concerning height and orifice 2D area (cases 1 and 3).

- *Distances of annotations from the ground truth surface*

The point to surface distances were computed for all cases and all user annotations. Corresponding boxplots, showing minimum values, percentiles (25^th^, 50^th^ and 75^th^), mean, maximum and outlier values for all cases are shown in Fig. **1**.

***Fig. 1*** ***Distances boxplot.*** *Boxplot representation of distances of the segmented points from the ground truth surface for all the cases for all the users. rLAX6, rLAX9 and rLAX18: radial long axis with 6, 9 and 18 planes respectively*

The segmented points are shown together with the ground truth surface, for case 1 and 2 (case 3 in Figure 10). The color scale was chosen according to the min and max values found on all three cases.

***Fig. 2 Distances of segmented point from ground truth valve surface CASE 1.*** *Annotation points and ground truth of the annotation of all users in all the modalities of case 1. The points are color coded depending on the distance from the surface, the scale is set according to the minimum and maximum distance values found for all three cases*

***Fig. 3 Distances of segmented point from ground truth valve surface CASE 2.*** *Annotation points and ground truth of the annotation of all users in all the modalities of case 2. The points are color coded depending on the distance from the surface, the scale is set according to the minimum and maximum distance values found for all three cases*
